# Supplementary material for: Predictive power of non-identifiable models
Source: Sci Rep. 2023 Jul 10;13:11143. doi: 10.1038/s41598-023-37939-8 (PMC10333263; doi:10.1038/s41598-023-37939-8)
Supplement: Supplementary file 1 — Supplementary Figures. [file 41598_2023_37939_MOESM1_ESM.pdf]

Supplementary Information

featuring the article

Predictive power of non-identifiable models

by Frederic Grabowski, Paweł Nałęcz-Jawecki, and Tomasz Lipniacki

Contents

Appendix Figure S1. Stiff directions in the nominal model 2

Appendix Figure S2. Training with an inadequate protocol 3

Appendix Figure S3. Training an incorrect model 4

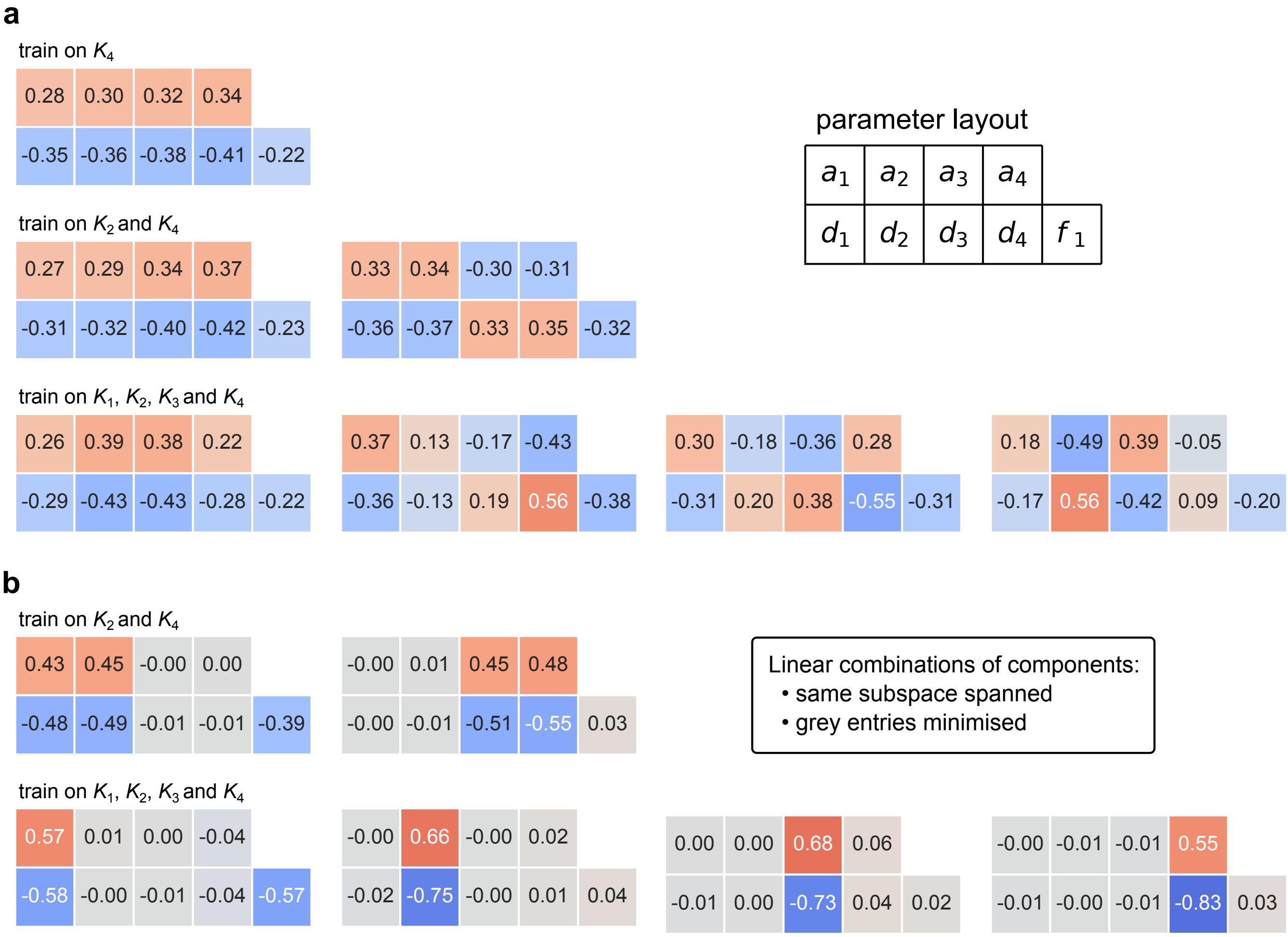

Figure S1. Stiff directions in the nominal model. (**a**) Principal components after training on K_4 (first row); K_2 and K_4 (second row); K1, K2, K3 and K4 (third row). Only components corresponding to stiff directions (with δ_i below 1.5 in Figure 2f) are shown. By construction, the principal components form an orthonormal basis, which spans the "stiff subspace". (**b**) Linear combinations of the principal components shown in (**a**), minimising the number of dominant entries while preserving the spanned "stiff subspace" and orthogonality. In this representation, one can easily interpret the stiff directions. For example, the first entry in the last row indicates that the value $a_1^{0.57}\, d_1^{-0.58}\, f_1^{-0.57}$ is well constrained when training on all model trajectories.

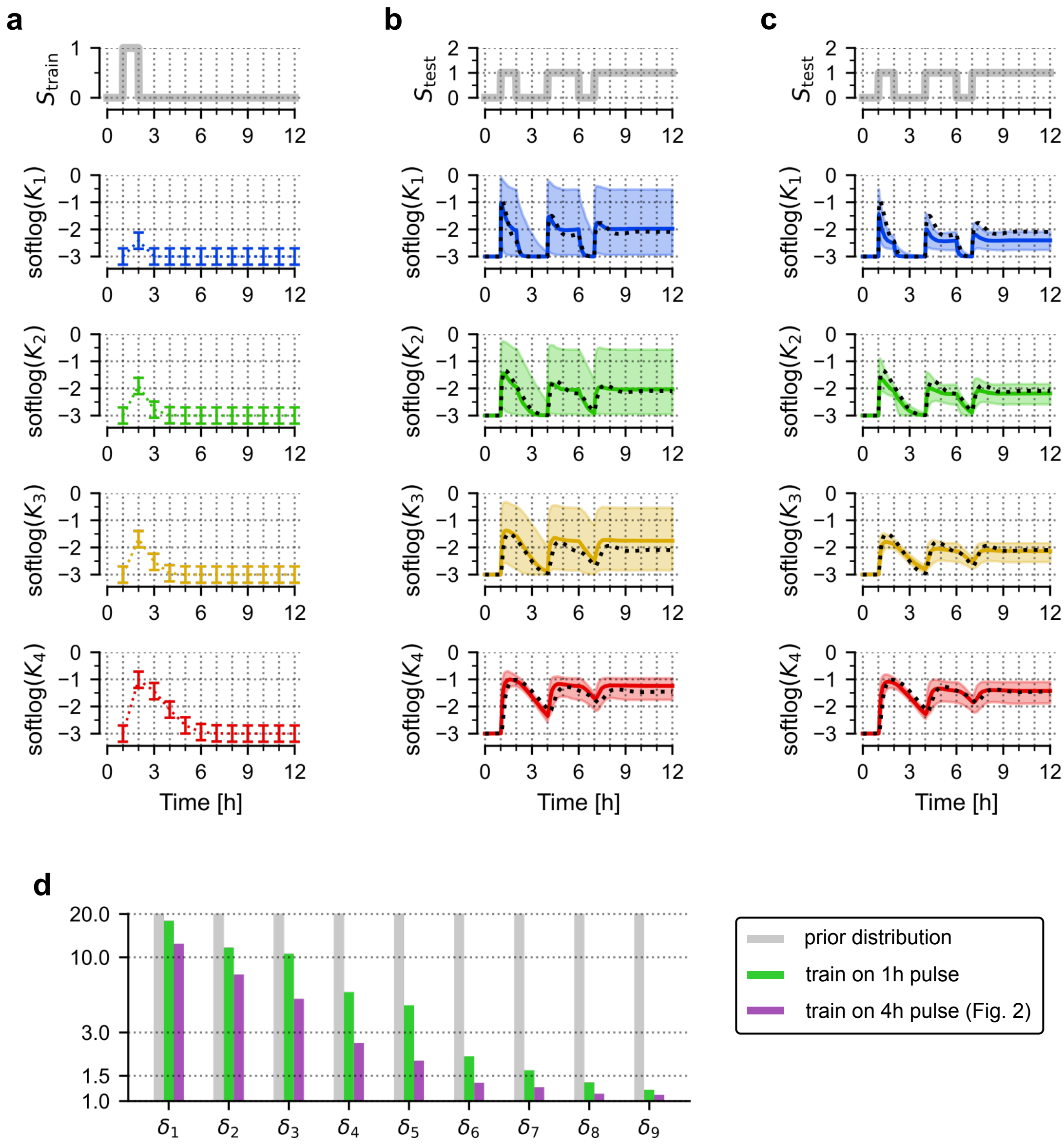

Figure S2. Training with an inadequate protocol. (**a**) Simulated trajectories of K_1, K_2, K_3, and K_4 in response to a shorter pulse (1h instead of 4h) S_{train} used for model training. Error bars show measurement errors assumed for the generation of the training data; and $\text{softlog}(x) = \log(0.001 + x)$. (**b**–**c**) Prediction of model responses to a test signal S_{test} after training on the trajectory of K_4 (**b**); and all four model variables (**c**). Black dotted lines show trajectories of the nominal model, coloured lines show (point-wise) medians of predictions, contours show 80% prediction bands. Including additional variables in training does not increase the model's predictive power for K_4. (**d**) Dimensionality reduction of the parameter space by training on all four model variables and different pulse lengths. Purple—training on a 4h pulse (repeated from Figure 2), green—training on a 1h pulse, grey—prior. Compared to training on a 4h pulse, principal multiplicative deviations are larger.

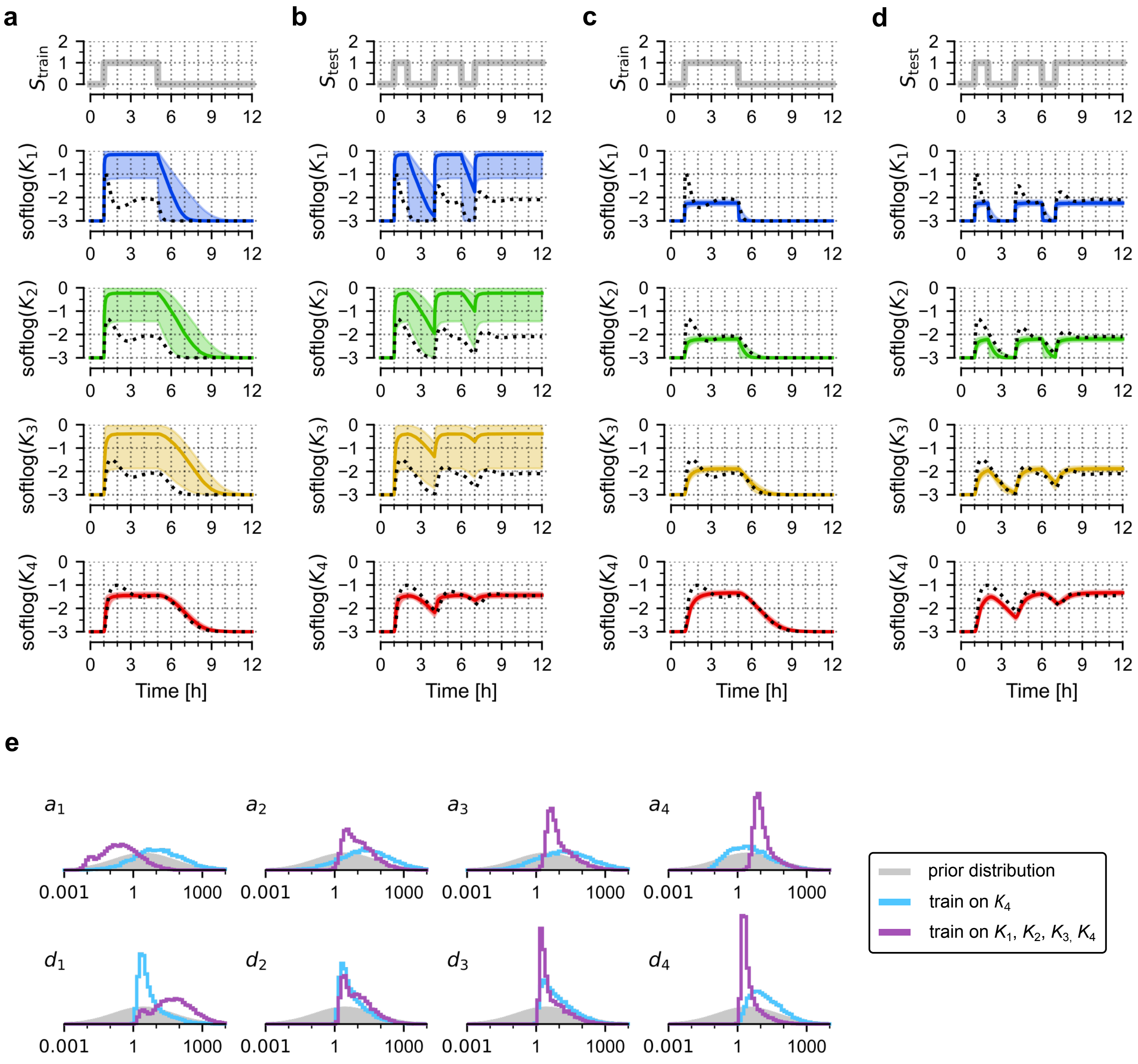

Figure S3. Training an incorrect model (without feedback). (**a**–**d**) Prediction of model responses to the train signal S_{train} (**a**, **c**) and a test signal S_{test} (**b**, **d**) after training on the trajectory of K_4 (**a**, **b**) and all four model variables (**c**, **d**); $\text{softlog}(x) = \log(0.001 + x)$. Black dotted lines show trajectories of the nominal model, coloured lines show (point-wise) medians of predictions, contours show 80% prediction bands. Model confident and wrong. (**e**) Histograms obtained from 10,000 samples generated from the posterior distribution of the incorrect model parameters after training on K_4 (blue); K_1, K_2, K_3 and K_4 (purple). Grey contours show prior distributions.
